# Bacterial Bloodstream Infections in HIV-infected Adults Attending a Lagos Teaching Hospital

**DOI:** 10.3329/jhpn.v28i4.6037

**Published:** 2010-08

**Authors:** Adeleye I. Adeyemi, Akanmu A. Sulaiman, Bamiro B. Solomon, Obosi A. Chinedu, Inem A. Victor

**Affiliations:** ^1^ Department of Microbiology, Faculty of Science, University of Lagos, Akoka, Nigeria; ^2^ Department of Haematology and Blood Transfusion, Lagos University Teaching Hospital, Idi-Araba, Nigeria; ^3^ Department of Obstetrics and Gynaecology, Bacteriology Research Laboratory, College of Medicine, University of Lagos, Lagos, Nigeria; ^4^ Institute of Child Health and Primary Care, Lagos University Teaching Hospital, Idi-Araba, Nigeria

**Keywords:** Bacteraemia, Bacterial infections, HIV, HIV infections, Nigeria

## Abstract

An investigation was carried out during October 2005–September 2006 to determine the prevalence of bloodstream infections in patients attending the outpatient department of the HIV/AIDS clinic at the Lagos University Teaching Hospital in Nigeria. Two hundred and one patients—86 males and 115 females—aged 14-65 years were recruited for the study. Serological diagnosis was carried out on them to confirm their HIV status. Their CD4 counts were done using the micromagnetic bead method. Twenty mL of venous blood sample collected from each patient was inoculated into a pair of Oxoid Signal blood culture bottles for 2-14 days. Thereafter, 0.1 mL of the sample was plated in duplicates on MacConkey, blood and chocolate agar media and incubated at 37 °C for 18-24 hours. The CD4+ counts were generally low as 67% of 140 patients sampled had <200 cells/μL of blood. Twenty-six bacterial isolates were obtained from the blood samples and comprised 15 (58%) coagulase-negative staphylococci as follows: *Staphylococcus epidermidis* (7), *S. cohnii cohnii* (1), *S. cohnii urealyticum* (2), *S. chromogenes* (1), *S. warneri* (2), *S. scuri* (1), and *S. xylosus* (1). Others were 6 (23%) Gram-negative non-typhoid *Salmonella* spp., *S.* Typhimurium (4), *S.* Enteritidis (2); *Pseudomonas fluorescens* (1), *Escherichia coli* (1), *Ochrobactrum anthropi* (1), *Moraxella* sp. (1), and *Chryseobacterium meningosepticum.* Results of antimicrobial susceptibility tests showed that coagulase-negative staphylococci had good sensitivities to vancomycin and most other antibiotics screened but were resistant mainly to ampicilin and tetracycline. The Gram-negative organisms isolated also showed resistance to ampicillin, tetracycline, chloramphenicol, and septrin. This study demonstrates that co-agulase-negative staphylococci and non-typhoidal *Salmonellae* are the most common aetiological agents of bacteraemia among HIV-infected adults attending the Lagos University Teaching Hospital, Nigeria. The organisms were resistant to older-generation antibiotics often prescribed in this environment but were sensitive to vancomycin, cefotaxime, cefuroxime, and other new-generation antibiotics.

## INTRODUCTION

Nigeria has the highest population in Africa, with 1 in 6 Africans being a Nigerian. The rate of HIV/AIDS prevalence has grown slowly from 1.9% in 1993 to 5.4% in 2003, and by the end of 2004, it was approaching 6% (1). However, a recent survey showed a decline to 4.6%, although the rate varies from state to state, with some recording as high as 10.6% (Benue state) and as low as 1.0% (Ekiti state) (2). The clinical manifestation of HIV secondary infections in developing countries, including Nigeria, shows a high prevalence of infections of the skin, gut, respiratory tract, tuberculosis, and malnutrition (3,4). The virus has a specific capacity to infect the CD4+ class of T lymphocyte, resulting in progressive decline in their (CD4+) number (5). This has serious health consequences since CD4+ cells constitute about 10% of the total T cell-pool. In AIDS patients, the number of CD4+ cells steadily decreases, and by the time opportunistic infections set in, CD4+ cells may be almost absent. These infections include spirochetal, protozoan, viral, fungal, mycobacterial and pyogenic bacterial infections (6). The later includes gastrointestinal diseases caused by enteric bacterial pathogens characterized by high rates of bacteraemia and frequent occurrence of diarrhoea.

Disseminated infections with *Salmonella* Typhimurium, *S.* Enteritidis, *S.* Arizona, *S.* Dublin, and other non-typhoidal *Salmonella* serotypes were recognized early in the HIV epidemic (7). Similarly, encapsulated bacteria, including *Streptococcus pneumoniae* and *Haemophilus influenzae* are two of the most common bacterial pathogens in HIV-infected persons (8). *Pseudomonas aeruginosa* has emerged as one of the most common causes of Gram-positive bacteraemia and pneumonia in HIV-infected hospitalized patients, and its incidence in AIDS patients appears to be on the rise, with many studies demonstrating an annual increase in cases (9).

Bacterial bloodstream infections constitute a significant public-health problem and present an important cause of morbidity and mortality in HIV-infected patients. A survey among HIV-I patients in Malawi showed that 30% had bloodstream infections. Organisms isolated were mainly *S. pneumoniae* (33%) and *Mycobacterium tuberculosis* (28%) (10). In a retrospective three-year study of all episodes of bloodstream infections in HIV-infected patients in Italy by Bonaldio *et al.* (11), the most frequent isolates were coagulase-negative staphylococci (n=33), *S. aureus* (n=7), *Pseudomonas* sp. (n=7), non-typhoid *Salmonella* (n=4), and fungi (n=1). Most patients had a CD4 count of <100.

To the best of our knowledge, there has been no previous study on bacterial bloodstream infections of teeming HIV-infected patients that abound in this country, apart from the limited survey carried out by Ogunsola *et al*. (12). The present study was conducted to determine the status of bacterial bloodstream infections in HIV-infected patients in this environment in relation to their CD4 T lymphocyte counts. This information will assist in identifying clinical predictors of bloodstream infections and in planning appropriate therapy.

## MATERIALS AND METHODS

### Study subjects

Patients were consecutive attendants at the HIV clinic of the Lagos University Teaching Hospital (LUTH). The LUTH is a tertiary-level teaching hospital and referral centre located in Lagos attending HIV-infected patients and others. The study was conducted during October 2005–September 2006.

The cohort consists of HIV-infected individuals referred to or identified at this hospital. The HIV-infected individuals included 86 males and 115 females aged 14-65 years. The entire subjects were attending the outpatient department of the HIV clinic.

The study was approved by the Medical Research and Ethics Committee of the Lagos University Teaching Hospital. Some patients were already on ART before the study commenced.

### Serological diagnosis of HIV

Serum samples obtained from the 201 patients were screened for HIV antibodies by enzyme-linked immunosorbent assay (ELISA) using Murex 1+2 kits (Murex Diagnostic, Dartfold, England). All sera found positive by ELISA were confirmed by Western blot (13).

### Determination of CD4

CD4 was estimated by micromagnetic bead method using kits supplied by Dynal Beads, UK. The procedure involved the addition of micromagnetic bead coated with anti-CD14 antibodies to whole blood. These magnetic beads bind to human monocytes that express anti-CD14 antigens. This is important so that these monocytes which equally express CD4 molecules are removed from the whole blood before quantitation of CD4+ cells. Following the depletion of blood monocytes, the second micromagnetic bead coated with anti-CD4 was added to the monocyte-depleted blood. The blood was placed in the magnetic field so that all the CD4 bearing cells in 100 μL of blood were attracted and isolated. These cells were resuspended in 100 μL of 5% glacial acetic acid tinted with Gentian violet and counted under the light microscope using Neubauer counting chamber.

### Collection and processing of blood samples

Venous blood samples (20 mL) were taken from each patient, and 10 mL were aseptically inoculated into a pair of Oxoid Signal blood culture bottles system asceptically. The blood samples were subsequently incubated for 2-14 days according to the standards of the World Health Organization (WHO) (14). Thereafter, 0.1 mL of the sample was drawn using a sterile syringe and plated out on MacConkey, blood and chocolate agar plates using the streak plate technique. Duplicate plates were inoculated for each of the sample. The plates were then incubated at 37°C for 18-24 hours and observed for bacterial growth. The chocolate agar plates were incubated under anaerobic conditions in an anaerobic jar for the possible isolation of microaerophiles.

### Characterization and identification of bacterial isolates

Colonies appearing on the agar plates were subcultured for purity, and a minimum of three colonies with identical morphology was selected individually and subjected to identification by standard biochemical tests (15). Finally, the isolates were confirmed using the API 20E and API staph-kits and were later identified using the API 20E (version 4.0), API20 WE (version 6.0), and API STAPH (version 4.0) software. Organisms identified as *Staphylococcus* were further subjected to coagulase test.

### Testing of antimicrobial sensitivity

Antimicrobial sensitivity test was carried out on each isolate using Oxoid single antibiotics discs, following the recommended standards of the National Committee on Clinical Laboratory Standards (16); 0.5 Macfarland standard of the pure culture of each isolate was inoculated into tryptose soya broth. Using sterile swab sticks, the resulting solution of each isolate was spread over plates of Mueller-Hinton agar until the surface became sticky. Coagulase-negative *Staphylococci* were screened for susceptibility to the following 17 antibiotics: ceftazidime (30 μg), cefotaxime (30 μg), ampicillin (10 μg), augmentin (30 μg), cefuroxime (30 μg), tetracycline (30 μg), chloramphenicol (30 μg), ofloxacillin (5 μg), piperacillin-tazobactam (P-100 μg, TZ-10 μg), penicillin (10 unit), oxacillin (1 μg), vancomycin (30 μg), septrin (25 μg), amikacin (30 μg), cloxacillin (5 μg), erythromycin (5 μg), and gentamicin (10 μg). The Gram-negative rods were screened for susceptibility to 12 of the listed drugs and nalidixic acid (30 μg). The appropriate antibiotic discs were placed on the lawn of bacterial isolates (4 discs per plate). The plates were incubated at 37°C for 24 hours after which the zones of inhibition were measured in mm using a table ruler. The results were recorded as susceptible, intermediate, or resistant. *E. coli* (NTCC 10148) and *S. aureus* (ATTCC 12600) were used as control.

## RESULTS

### Distribution of age

The study population comprised 201 HIV patients—86 males and 115 females. They were aged 14-65 years with a mean age of 34.3 [standard deviation (SD) 9.9] years (Table 1). Of them, 143 (71.5%) were aged 20-40 years. Teenagers and people in the fifth decade seemed to share identical affliction rate (6%) while those in their fourth decade were more frequently affected (17.5%). The figure shows the number of study patients when they were first diagnosed HIV-positive.

**Table 1.** Age and sex distribution of 201 HIV/AIDS patients studied

Age-group (years)	Male (n=86)	Female (n=115)	Total (n=201)	Total %
11-20	2	10	12	6.0
21-30	26	44	70	34.8
31-40	33	40	73	36.3
41-50	18	15	33	16.4
51-60	6	6	12	6.0
>60	1	-	1	0.5

### CD4 range, sex, and antiretroviral status

Table 2 shows the CD4 count range, antiretroviral therapy, and bloodstream infections of 140 of the 201 patients. Ninety-four (67.1%) patients had a CD4 count of <200 cells/μL of blood. Seventeen (65%) of 26 patients positive for bloodstream infections belonged to this group. The majority (69.6%) of the patients with CD4 counts above the 200 cells/μL threshold were on antiretroviral therapy. All the patients but one who recorded >500 cells/μL were female.

**Table 2.** CD4 count range, ART and BSI status of 140* HIV-positive patients attending Lagos University Teaching Hospital

CD4 range (cells/uL of blood)	Male	Female	Total	%	No. on ART (%)	No. not on ART (%)	No. positive for BSI (%)
<20	9	8	17	12.1	0 (0)	17 (25.0)	6 (23.1)
21-100	22	20	42	30.0	19 (26.3)	23 (33.8)	7 (26.9)
101-200	22	13	35	25.0	21 (29.2)	14 (20.6)	4 (15.4)
201-500	16	19	35	25.0	24 (33.3)	11 (16.2)	8 (30.8)
501-1,000	1	8	9	6.4	6 (8.3)	3 (4.4)	1 (3.8)
>1,000	-	2	2	1.4	2 (2.8)	0 (0)	0 (0)
Total	70	70	140	100.0	72 (100)	68 (100)	26 (100)

*61 patients have not done their CD4 count at the time of recruitment into the study;

ART=Antiretroviral therapy;

BSI=Bloodstream infection;

HIV=Human immunodeficiency virus

### Bacterial isolates

Twenty-six bacterial isolates comprising mainly 15 coagulase-negative staphylococci (58%) and six non-typhoidal *Salmonellae* (23%) were isolated from the blood samples of 26 (12.9%) of the 201 patients (Table 3). There was an unusual isolation of *Ochrobactrum anthropi* (n=1), Moraxella sp. (n=1), and *Chryseobacterium meningospecticum* (n=1) from three patients. Other isolates were *E. coli* (n=1) and *P. aeruginosa* (n=1). Altogether, 26 patients—17 females and nine males—had bloodstream bacterial infections.

**Table 3.** CD4 counts, antiretroviral treatment status, and bacteria isolated from 26 HIV-positive patients attending Lagos University Teaching Hospital

Sl. no.	Bacterial isolate	CD4 count cells/μL of blood	ART status	Sex of patients
1	*Staphylococcus scuri*	224	+	F
2	*Staphylococcus cohni urealiticum*	221	+	F
3	*Staphylococcus epidermidis*	191	+	M
4	*Salmonella* Typhimurium	55	-	M
5	*Staphylococcus warneri*	145	+	F
6	*Staphylococcus epidermidis*	288	+	M
7	*Staphylococcus epidermidis*	34	-	M
8	*Staphylococcus xylosus*	62	-	F
9	*Staphylococcus chromogenes*	10	-	F
10	*Staphylococcus cohni cohni*	18	-	F
11	*Staphylococcus epidermidis*	460	+	F
12	*Salmonella* Enteritidis	15	-	F
13	*Salmonella* Enteritidis	146	-	M
14	*Staphylococcus cohni urealiticum*	60	-	F
15	*Staphylococcus epidermidis*	6	-	F
16	*Staphylococcus epidermidis*	604	+	F
17	*Staphylococcus warneri*	46	-	M
18	*Salmonella* Typhimurium	392	+	F
19	*Salmonella* Typhimurium	14	-	M
20	*Staphylococcus epidermidis*	7	-	M
21	*Salmonella* Typhimurium	47	-	M
22	*Escherichia coli*	360	+	F
23	*Pseudomonas flourescence*	228	+	F
24	*Ochrobactrum anthropi*	220	+	F
25	*Moraxella* sp.	110	-	F
26	*Chryseobacterium meningospecticum*	88	-	M

ART=Antiretroviral therapy;

F=Female;

HIV=Human immunodeficiency virus;

M=Male

### Antimicrobial susceptibility patterns

Table 4 shows the antimicrobial susceptibility patterns of the staphylococci isolates to 17 antimicrobial agents while Table 5 shows those of the Gram-negative rods to 13 drugs. The coagulase-negative staphylococci demonstrated good susceptibility patterns to oxacillin and vancomycin. The Gram-negative rods also demonstrated good sensitivity patterns to ceftazidime, cefotaxime, augmentin, cefuroxime, and other antibiotics. Most staphylococci isolates were, however, resistant to ampicillin (n=11, 73.3%), tetracycline (n=8, 53%), and penicillin G (n=11, 73.3%). The resistance pattern varied from single (TET or P) to sextiple (AMP-TET-CM-OFX-P-CN) observed in one *S. epidermidis* isolate. The Gram-negative rods, on the other hand, were resistant to ampicillin (n=7, 63%), tetracycline (n=7, 63%), chloramphenicol (n=7, 63%), and septrin (63%). They showed a pattern varying from single (TET or CAZ) to sextiple (AMP-AMC-TET-CM-OFX-SXT). The latter was encountered in the *E. coli* isolate.

**Fig.**
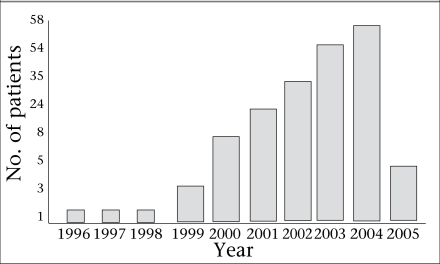
Distribution of HIV-infected patients at first diagnosis at Lagos University Teaching Hospital

**Table 4.** Antimicrobial susceptibility patterns of staphylococci isolates causing bloodstream infections in HIV-positive patients in Lagos

Identification	CAZ	CTX	AMP	AMC	CXM	TET	C	OFX	TZP	P	OX	VA	SXT	AK	OB	E	CN	Resistance patterns
*Staphylococcus epidermidis*	S	I	R	S	S	R	S	S	S	R	S	S	R	S	S	S	R	AMP-TET-P-SXT-CN
*S. epidermidis*	S	I	R	S	S	R	S	S	S	R	S	S	I	S	S	I	R	AMP-TET-P-CN
*S. epidermidis*	S	S	R	S	R	R	R	S	S	R	S	S	S	S	S	S	I	AMP-CXM-TET-CM-P
*S. epidermidis*	S	S	R	S	R	R	R	S	S	S	S	S	I	S	S	S	R	AMP-CXM-TET-CM-CN
*S. epidermidis*	S	S	R	S	S	S	S	S	S	R	S	S	S	S	S	I	S	AMP-P
*S. epidermidis*	S	S	R	S	S	S	S	S	S	R	S	S	S	S	S	S	S	AMP-P
*S. epidermidis*	S	I	R	S	S	R	R	R	S	R	S	S	S	S	S	S	R	AMP-TET-CM-OFX-P-CN
*S. cohnii cohnii*	R	I	R	S	I	R	S	S	S	R	S	S	S	S	S	I	S	CAZ-AMP-TET-P
*S. cohnii urealyticum*	I	I	R	S	S	S	S	S	S	R	S	S	S	S	S	S	S	AMP-P
*S. cohnii urealyticum*	I	S	S	S	S	S	S	S	S	R	S	S	S	S	S	S	S	P
*S. chromogenes*	S	S	S	S	S	R	S	S	S	S	S	S	S	S	S	I	S	TET
*S. warneri*	S	S	S	S	S	S	S	S	S	S	S	S	S	S	S	I	S	-
*S. waneri*	S	S	R	S	S	S	S	S	S	R	S	S	S	S	S	S	S	AMP-P
*S. sciuri*	I	S	S	S	R	R	S	S	S	S	S	S	S	S	S	I	S	CXM-TET
*S. xylosus*	R	S	R	S	S	S	R	S	S	R	S	S	S	S	S	S	S	CAZ-AMP-CM-P

AK=Amikacin;

AMP=Ampicillin;

AMC=Augmentin;

CAZ=Ceftazidime;

CM=Chloramphenicol;

CN=Gentamicin;

CXM=Cefuroxime;

CTX=Cefotaxime;

E=Erythromycin;

I=Intermediate;

OB=Cloxacillin;

OFX=Ofloxacin;

OX=Oxacillin;

P=Penicillin;

R=Resistant;

S=Sensitive;

SXT=Septrin;

TET=Tetracycline;

TZP=Piperacillin-tazobactam;

VA=Vancomycin

**Table 5.** Antimicrobial susceptibility patterns of the Gram-negative rods causing bloodstream infections in HIV-infected patients in Lagos

Identification	CAZ	CTX	AMP	AMC	CXM	TET	CM	OFX	TZP	SXT	AK	NAL	CN	Antibiotic susceptibility patterns
*Salmonella* Typhimurium	S	S	R	S	I	R	R	S	S	R	S	S	S	AMP-TET-CM-SXT
*Salmonella* Typhimurium	S	S	R	S	S	R	R	S	S	R	S	S	S	AMP-TET-CM-SXT
*Salmonella* Typhimurium	S	S	R	S	S	R	R	S	S	R	S	S	S	AMP-TET-CM-SXT
*Salmonella* Typhimurium	S	S	R	S	S	S	R	S	I	S	S	S	S	AMP-CM
*Salmonella* Enteritidis	S	S	R	S	S	R	S	S	S	R	S	S	S	AMP-TET-SXT
*Salmonella* Enteritidis	S	S	R	S	S	I	R	S	S	R	S	S	S	AMP-CM-SXT
*Escherichia coli*	S	S	R	R	S	R	R	R	I	R	S	S	S	AMP-AMC-TET CM-OFX-SXT
*Pseudomonas flourescens*	R	I	S	S	S	S	S	I	S	S	S	S	S	CAZ
*Chryseobacterium menlngospecticum*	S	S	S	R	S	R	R	S	I	R	S	S	S	AMC-TET-CM-SXT
*Moraxella* sp.	S	S	I	S	S	R	S	S	S	S	S	S	S	TET
*Ochrobacter anthropi*	R	R	S	S	S	I	S	S	I	S	S	R	S	CAZ-CTX-NAL

AK=Amikacin;

AMC=Augmentin;

AMP=Ampicillin;

CAZ=Ceftazidime;

CM=Chloramphenicol;

CN=Gentamicin;

CTX=Cefotaxime;

CXM=Cefuroxime;

I=Intermediate;

NAL=Nalizidic acid;

OFX=Ofloxacin;

R=Resistant;

S=Sensitive;

SXT=Septrin;

TET=Tetracycline;

TZP=Piperacillin-tazobactam

## DISCUSSION

The age range of 14-65 years with a mean of 34 years of HIV patients attending the LUTH was observed to be in line with the general picture of HIV/AIDS infection worldwide, especially in Africa. It has been noted that the pandemic affects mainly sexually-active age-groups (3,17). There were more females than males in the study subjects (115:86), and this is a reflection of disproportionate impact of HIV on women and girls than on men. Obi *et al*. established that there were more HIV-positive females than males in studies in South Africa, indicating a ‘gender bias’ (18). The vulnerability of women to HIV in sub-Saharan Africa stem from their greater physiological susceptibility and the severe social, legal and economic disadvantages that confront them (19). Our study also confirmed the upsurge in reported cases of HIV infection in Nigeria until 2005 when a noticeable decline in infection rate began (2). It was observed in this study that the majority (67%) of the patients had CD4 counts of <200 cells/μL of blood, and this was more pronounced among subjects who were not yet on antiretroviral therapy. Among the later group were five subjects whose CD4 counts were <10 cells/μL. This observation is in agreement with already-established knowledge of HIV pathogenesis which indicates that there is a steady decline in the CD4 counts of patients and by the time opportunistic infections set in there may be no more CD4 cells present in the immune system (5). The findings of our study also showed that 48.5% of the subjects had no access to antiretroviral treatment. Some of them had been diagnosed HIV-positive as far back as 2000 and 2001 respectively. This lack of access to medical care, especially the much-needed antiretroviral drugs in Africa, has been widely reported by the WHO and other workers (17). Although this problem is now being addressed in Nigeria at the governmental level, it will take some time before every patient in need will be able to access antiretroviral therapy. Another disturbing fact is that some patients who have been on antiretroviral therapy for a fairly long time had low CD4 counts. This could be attributed to non-adherence to treatment regime and also lack of proper dietary balance. It is known that the many subjects belonged to the low-income group (itenary traders and artisans) and probably could afford the cost of the drugs and nourishable foods which are important ingredients for boosting the population of T cells in their blood. However, the majority of the patients on antiretroviral therapy had their (CD4) counts above the 200 cells/μL threshold, and nearly all the patients who recorded >500 cells/μL were female.

Bloodstream infections have long been observed to appear frequently in HIV/AIDS patients. Bekele *et al.* reported that, of 1,225 hospitalized patients at the University of Florida Health Science Center, 88 (7%) had bloodstream infections, and 73 of the infections were community-acquired (20). Comparatively, same workers recorded a bacterial infection rate of 12% in patients with HIV when blood of 201 patients were cultured. Ogunsola *et al*. reported the infection rate of 33% in 67 AIDS patients attending the LUTH (12). In the present study, a prevalence of 12.9% was recorded. In 2000, Ippolito *et al*. also recorded 12.1% cases of bloodstream infections in a prospective multicentre study in HIV patients with advanced stage of the disease (21). Similarly, 30% prevalence rate of bloodstream infections was recorded in Malawian patients (10).

Bloodstream infections constitute a significant public-health problem and represent an important cause of morbidity and mortality in HIV/AIDS patients. In our study, coagulase-negative Gram staphylococci dominated the number of bacteria isolated as 15 (57.7%) of the 26 isolates belonged to this group. Gooze and Choi *et al*. had established that coagulase-negative staphylococci are a major cause of infection in immuno-compromised patients (22,23). Similarly, Bonadio *et al*. reported that the most frequently-isolated bacteria were coagulase-negative staphylococci (11), and *Staphylococcus cohnii* had been implicated in community-acquired pneumonia in an HIV-infected patient (24). Other organisms encountered in our study included non-typhoid *Salmonellae* (n=6), *P. fluorescens* (n=1), *and E. coli* (n=1). Bonadio *et al*. also established the presence of non-typhoid *Salmonellae, Pseudomonas* sp., and one fungus in 68 blood samples from HIV-infected patients with bloodstream infections (11). An increase in the frequency and severity of non-typhoid *Salmonella* has been reported with AIDS, and infections often present as recurrent diarrhoea with relapsing bacteraemia (11,12,25). *S*. Typhimurium and *S.* Enteritidis are the two most common serotypes isolated from blood of patients with AIDS in the United States (26) and Africa (7,27). This is in agreement with the findings of our study. Invasive non-typhoid *Salmonellae* are endemic in sub-Sahara Africa, and it has been postulated that transmission between humans—both within and outside health facilities (community-acquired)—may be important (28). *P. aerugnosa* and other *Pseudomonas* have also been more frequently recognized as nosocomial bacteraemia in HIV-infected patients (29) and were reported to account for 12% in hospital patients in Italy (30). Only one of the blood samples cultured grew *E. coli*. This organism is not frequently isolated in cases of bloodsteam infections among HIV/AIDS patients, and of 861 blood samples examined by Ippolito *et al*. (21), only seven were positive for *E. coli*. An unusual occurrence was the isolation of *O. anthropi, C. meningosepticum,* and *Moraxella* sp. Early reports on bloodstream infections had documented the prevalence of encapsulated bacteria and non-typhoid *Salmonellae* but, during the recent years, broader aetiological spectrum has become evident. The first two cases of *O. anthropi*-associated septicaemia occurring in patient with HIV-infected disease were reported about a decade ago (31). Similarly, although the role of *C. meningosepticum* in immunocompromised host has been recognized (32), clinical data detailing this infection remain limited. Hung *et al*. reported that the presence of central venous line infection was associated with a poor outcome in patients with *C. meningosepticum* (33). According to Manfredi *et al*., *M. catarrhalis* may be responsible for appreciable morbidity among patients with advanced HIV infections with low CD4 counts (34).

Results of antimicrobial susceptibility tests revealed that most coagulase-negative staphylococci were susceptible to most antibiotics screened. Specifically, none was resistant to methicillin and vancomycin. Some of them were resistant to ampicillin, tetracycline, and penicillin. This is in line with results of previous studies. Shittu *et al*. reported that all 200 isolates of *S. aureus* from clinical samples were susceptible to teicoplanin, vancomycin, fusidic acid, and rifampicin but showed high resistance to penicillin, sulphonamides, and tetracycline (35). Taiwo *et al*. also reported cumulative resistance of 38-85% to chloramphenicol, tetracycline, ampicillin, gentamycin, and penicillin G among bacterial isolates from bloodstream infections in a Nigerian university teaching hospital (36). The isolated Gram-negative bacteria also showed good sensitivity patterns to ceftazidin, cefotaxime, augmentin, and cefuroxime, indicating that they do not produce extended betalactamase. However, they too showed varying resistance to older-generation antibiotics, such as ampicillin, tetracycline, chloramphenicol, and septrin. The multi-drug resistance patterns observed in this study are similar to those earlier reported by Adeleye and Adesoye (37) where septiple resistance comprising SXT-CT-FD-S-C-TET-AM was found to dominate in clinical isolates studied. Similarly, Taiwo *et al*. observed seven patterns of antibiotic resistance, with resistance comprsing the earlier-mentioned antibiotics (AMP-TET-C-GM-P) being most common in bloodstream bacterial isolates (36).

The present study, thus, established that bacterial bloodstream infection is a common occurrence in HIV-positive patients attending the LUTH. The prevalence is comparable with what was obtained in the developed world but less than what is observed in the South African region. The majority of the study patients with bloodstream infection had low CD4 counts, and the antibiotic resistance profile of the bacteria indicate that these are community-acquired. Therefore, a continuous surveillance and intervention strategies should be put in place to manage cases of bloodstream infections in HIV-positive patients in Nigeria.

## ACKNOWLEDGEMENTS

The study was funded by the Central Research Grant, University of Lagos, Akoka, Lagos (Grant No. CRC 2001/19b).
